# Zika Virus–Associated Birth Defects, Costa Rica, 2016–2018

**DOI:** 10.3201/eid2702.202047

**Published:** 2021-02

**Authors:** Adriana Benavides-Lara, María de la Paz Barboza-Arguello, Mauricio González-Elizondo, Marcela Hernández-deMezerville, Helena Brenes-Chacón, Melissa Ramírez-Rojas, Catalina Ramírez-Hernández, Nereida Arjona-Ortegón, Shana Godfred-Cato, Diana Valencia, Cynthia A. Moore, Alejandra Soriano-Fallas

**Affiliations:** Instituto Costarricense de Investigación y Enseñanza en Nutrición y Salud, Cartago, Costa Rica (A. Benavides-Lara, M.P. Barboza-Arguello, M. González-Elizondo);; Caja Costarricense del Seguro Social, San José, Costa Rica (M. Hernández-deMezerville, H. Brenes-Chacón, C. Ramírez-Hernández, N. Arjona Ortegón, A. Soriano-Fallas);; Ministry of Health, San José (M. Ramírez-Rojas);; Centers for Disease Control and Prevention, Atlanta, Georgia, USA (S. Godfred-Cato, D. Valencia, C.A. Moore)

**Keywords:** Birth defects, Zika virus, prevalence, surveillance, viruses, congenital infections, microcephaly, Costa Rica

## Abstract

After Zika virus (ZIKV) infection in Costa Rica was confirmed in January 2016, the national surveillance system was enhanced to monitor associated birth defects. To characterize the ZIKV outbreak among live-born infants during March 2016–March 2018, we conducted a descriptive analysis. Prevalence of ZIKV-associated birth defects was 15.3 cases/100,000 live births. Among 22 infants with ZIKV-associated birth defects, 11 were designated as confirmed (positive for ZIKV) and 11 were designated as probable cases (negative for ZIKV or not tested, but mother was expsed to ZIKV during pregnancy). A total of 91% had microcephaly (head circumference >2 SDs below mean for age and sex), 64% severe microcephaly (head circumference >3 SDs below mean for age and sex), 95% neurodevelopmental abnormalities, 82% brain anomalies, 41% eye abnormalities, and 9% hearing loss. Monitoring children for >1 year can increase identification of ZIKV-associated abnormalities in addition to microcephaly.

## Abstract 2

Enhanced birth defect surveillance increased identification of virus-associated abnormalities, including microcephaly.

Zika virus (ZIKV) is an RNA virus of the Flaviviridae family and is transmitted primarily by mosquitos of the genus *Aedes* (*Stegomyia*). The virus was discovered in Uganda in 1947 (*1*) and isolated from humans in Nigeria in 1953; in subsequent years, small clusters of infection in humans from Africa and Asia were reported (*2*). ZIKV outbreaks were identified in Yap in 2007 and in French Polynesia in 2013–2014 (*2*–*4*). In 2015, ZIKV reached the continental Americas, and an outbreak in Brazil was identified (*5*). In September 2015, an increased number of children were born with microcephaly and other central nervous system (CNS) defects in countries where ZIKV was circulating (*6*–*8*).

An article published in early 2017 described the most severe phenotype of ZIKV-associated birth defects (ZBD) (*9*). The 5 key characteristics of that phenotype are severe microcephaly with collapse of the skull and redundancy of the scalp consistent with the fetal brain disruption sequence (*10*), thinning of the cerebral cortex and subcortical calcifications, macular scarring with focal retinal pigment mottling, congenital joint contractures, and hypertonia with symptoms of extrapyramidal involvement. These findings were noted to be more characteristic of ZIKV infection than other congenital infections; however, they did not constitute a case definition. Several subsequent studies supported that these defects were associated with ZIKV infection during pregnancy (*11*–*13*).

In Costa Rica, microcephaly has been monitored by the national birth defects surveillance system (NBDSS) since 1985. Before the ZIKV epidemic, Costa Rica was among countries with the highest prevalence of microcephaly in Latin America; microcephaly prevalence during 2011–2015 was 4.2 cases/10,000 live births (95% CI 3.6–4.9 cases/10,000 live births; n = 153; annual median 31) (*14*). In January 2016, the National Virology Reference Center (Cartago, Costa Rica), in coordination with the NBDSS, implemented laboratory-based surveillance for ZIKV disease; in February 2016, the NBDSS, along with health authorities, initiated ZBD surveillance (Figure 1). To characterize the effects of the Zika virus outbreak on live-born infants, we reviewed enhanced surveillance data for birth defects and the clinical characteristics of infants with confirmed and probable ZBD born in Costa Rica during March 2016–March 2018. In accordance with the Costa Rican Biomedical Research Legislation, Article 7, the analysis of surveillance data was registered in the National Council of Health Investigation.

**Figure 1:**
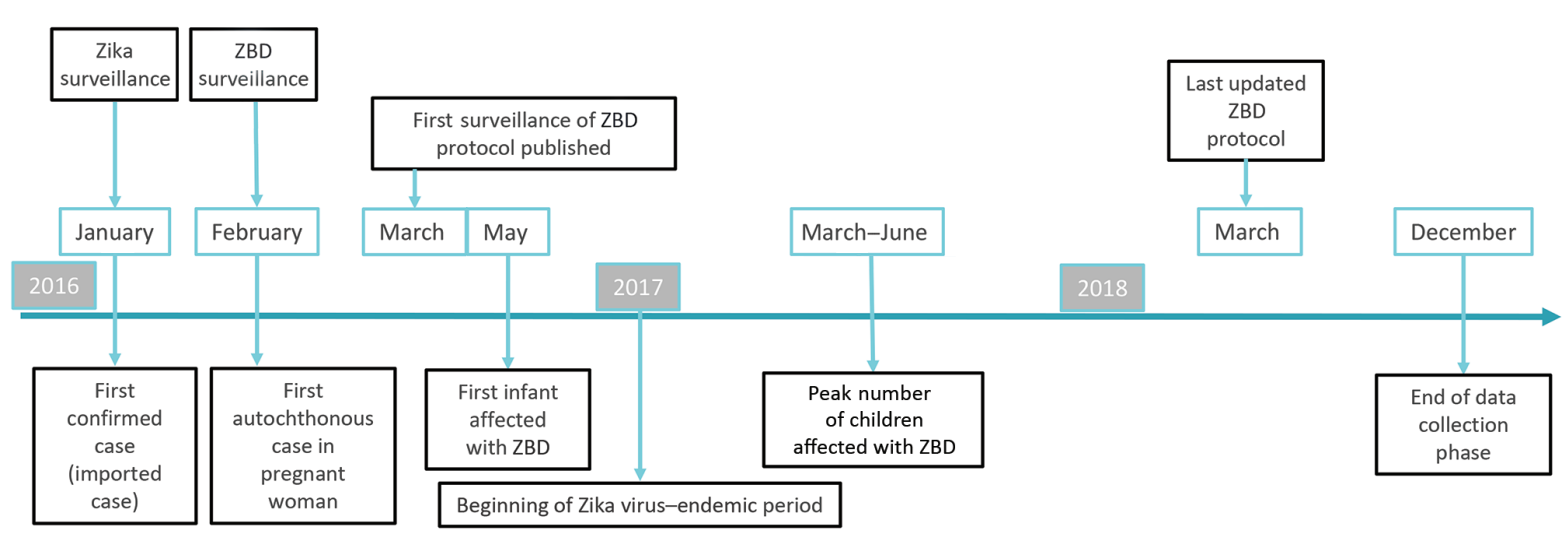
Key events involving ZBD surveillance, Costa Rica, March 2016–March 2018. In Costa Rica, laboratory testing using real-time reverse transcription PCR was implemented in late January 2016 (*15*–*17**)*. Although the first autochthonous case in Costa Rica was detected in a pregnant woman in February 2016 (*16*), a case was published in the United States about a traveler infected in December 2015 in Costa Rica (*17*). ZBD, Zika virus–associated birth defects.

## Methods

### Birth Defects Surveillance System

We conducted a descriptive analysis based on retrospective data collected for the NBDSS during the study period. The Costa Rican Birth Defects Register Center is an NBDSS that collects information on internal and external ZBD for all live-born infants up to 1 year of age. Passive reporting is mandatory for public and private hospitals; live-birth coverage is 96%. In February 2016, birth defect surveillance was enhanced by creation of a protocol that established the follow-up of cases, including laboratory tests for ZIKV and other differential diagnoses (*18*). Cases reported to the NBDSS were reviewed and classified by a multidisciplinary team in Costa Rica and by subject matter experts from the US Centers for Disease Control and Prevention.

### Microcephaly

Microcephaly is defined as head circumference measurement >2 SDs below the mean for a given age and sex (*19*); it is severe when the circumference is >3 SDs below the mean for a given age and sex. For infants born at term, we used World Health Organization growth charts (*20*). For preterm infants, microcephaly was defined as head circumference below the third percentile according to the Fenton Growth Charts (https://live-ucalgary.ucalgary.ca/resource/preterm-growth-chart/preterm-growth-chart). Congenital and postnatal-onset microcephaly were included.

### Suspected Cases

All potential cases of birth defects were reported to NBDSS. These reports were reviewed to determine whether they met the criteria for a suspected case of birth defects. Suspected cases were those that met >1 of the following criteria:

• Live-born infants with microcephaly, regardless of laboratory findings in the mother or infant, or maternal symptoms of ZIKV (rash and fever) during pregnancy

- Any live-born infant with >2 of the following findings (or 1 + microcephaly), regardless of laboratory findings in the mother or infant or maternal ZIKV symptoms during pregnancy:
  - CNS: intracerebral calcifications, cerebellar hypoplasia, thinning of the cerebral cortex, corpus callosum anomalies, ventriculomegaly or increased extra-axial fluid, abnormal pattern of cerebral gyri (e.g., polymicrogyria, lissencephaly), and specific neurodevelopmental findings (e.g., psychomotor development delay, spasticity, persistent irritability, seizures, swallowing disorders, movement abnormalities, or extrapyramidal changes)
  - Sensorineural deafness
  - Eye: structural abnormalities (e.g., microphthalmia, coloboma, cataracts or intraocular calcifications; posterior pole anomalies such as chorioretinal atrophy, optic nerve abnormalities, focal retinal pigment mottling)
  - Arthrogryposis or multiple joint contractures affecting >1 major joint or talipes equinovarus
- Live-born infants without microcephaly but with any major birth defect not consistent with a ZBD (e.g., significant cardiac defect) or with specific neurodevelopmental findings mentioned above, born to a mother with probable or confirmed ZIKV infection during pregnancy (defined as a mother with symptomatic ZIKV infection during pregnancy and/or a strong epidemiologic link to ZIKV during pregnancy [lives in high ZIKV–endemic area or has close contact with ZIKV–positive person] with or without positive laboratory test result for ZIKV during pregnancy)

For all suspected case-patients, a serum sample, urine sample, or both were collected from the infant before hospital discharge. Samples were tested for ZIKV RNA by using established singleplex real-time reverse transcription PCR (rRT-PCR) (*3*). Serum was tested in parallel with Zika IgM Antibody Capture ELISA (*3*,*21*). The NBDSS was immediately notified, the case was reviewed, and the infant was referred to a pediatrician and the congenital infection clinic (CIC). A multidisciplinary assessment of the child was conducted and included evaluation by pediatricians and the CIC; laboratory testing for syphilis, toxoplasmosis, rubella, and cytomegalovirus infection; cranial ultrasonography; and indirect ophthalmologic examination and neonatal auditory screening by otoacoustic emissions, followed by auditory brainstem response. Referral to a geneticist, pediatric cardiologist, neurodevelopmental specialist, or pediatric neurologist was dependent on examination findings. Thus, some of the children underwent complementary testing such as chromosomal or fluorescence in situ hybridization analysis, cardiac or abdominal ultrasonography, computerized tomography, and specialized neurodevelopmental assessments.

### Classification and Characterization of Suspected Cases

**Figure 2:**
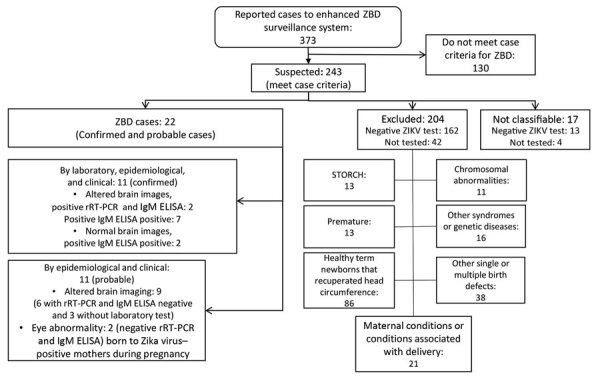
Reported cases and classification of suspected cases of ZBD according to protocol, Costa Rica, March 2016–March 2018. rRT-PCR, real-time reverse transcription PCR; STORCH, syphilis, toxoplasmosis, rubella, cytomegalovirus, and hepatitis B (note that Costa Rica does not include hepatitis B in its standard evaluations); ZBD, Zika virus–associated birth defects.

All clinical, epidemiologic, and laboratory data for suspected cases were reviewed. Cases were classified as confirmed, probable, excluded, and not classifiable (Figure 2) as follows:

• Confirmed case-patients were infants with ZBD (clinical criteria) for whom a sample taken before hospital discharge was positive for ZIKV by rRT-PCR or IgM ELISA (laboratory criteria) and who had an epidemiologic link (mother with ZIKV symptoms or was ZIKV positive by rRT-PCR during pregnancy or was from a highly ZIKV–endemic community).

• Probable case-patients were infants with ZBD (clini­cal criteria) who had negative ZIKV results by rRT-PCR or IgM ELISA or were not tested but whose mother had laboratory-confirmed ZIKV infection or symptoms compatible with ZIKV infection or had an epidemiologic link, and no other cause for the birth defect was identified.

• Excluded case-patients were infants with ZBD not related to ZIKV infection, who had negative laboratory results for ZIKV by rRT-PCR and IgM ELISA or were not tested and whose mother had negative ZIKV results, no symptoms of ZIKV infection, or no clear exposure to ZIKV during pregnancy. Excluded cases also included infants who had other known etiologies for microcephaly or the birth defect or had a presumed syndrome of undetermined cause. This group also included infants with a diagnosis of microcephaly at birth whose head circumference by 1 year of age was <2 SDs below the mean (and did not have any other birth defects).

• Not classifiable case-patients were in infants with insufficient information to be appropriately included in the previous categories.

### Analysis

We calculated population-based birth prevalence and 95% CI for confirmed and probable cases of ZBD and microcephaly during the period of enhanced surveillance and compared the prevalence ratio for microcephaly with the baseline prevalence during 2011–2015. We also calculated the distributions of specific birth defects and neurodevelopmental abnormalities among infants with ZBD. Total births for the period were obtained from the National Institute of Statistics and Censuses (http://www.inec.go.cr). To characterize infants with ZBD, we used the mother’s province of origin; mother’s history of exposure to ZIKV during pregnancy (associated symptoms or laboratory confirmation); and the infant’s head circumference, weight, length, gestational age, ZIKV molecular and serologic test results, other reported birth defects, and neurodevelopmental anomalies.

## Results

Of 373 potential cases reported to the NBDSS, 243 met the criteria for a suspected case (Figure 2); 150 (62%) of the 243 infants were female. Compared with microcephaly baseline data for Costa Rica (*22*), the birth prevalence of microcephaly increased from 4.2 (95% CI 3.6–4.9) cases/10,000 live births during 2011–2015 to 15.5 (95% CI 13.5–17.5) cases/10,000 live births during the Zika outbreak (March 2016–March 2018); prevalence ratio was 3.7 (95% CI 3.0–4.5).

### Evaluation of Suspected ZBD Cases

A pediatric infectious disease specialist at the CIC examined 40% (96/243) of the infants with suspected ZBD >1 time; a pediatrician evaluated the rest. The most frequent birth defect was microcephaly; 88% (213/243) had microcephaly at birth. Among those, 26% (55/213) had severe microcephaly. Postnatal-onset microcephaly developed in 5% (12/243), and no microcephaly but other criteria that met the definition of suspected ZBD was found for 7% (18/243). A total of 9% (22/243) of suspected cases-infants were classified as having confirmed or probable ZBD, 84% (204/243) were excluded, and 7% (17/243) were not classifiable (Table 1; Figure 2). 

**Table 1:** Distribution of 204 excluded cases according to exclusion criteria for Zika virus–associated birth defects, Costa Rica, March 2016–March 2018*

Category, cause	No. cases
Chromosome anomaly, n = 11	
Ring chromosome 4. Possible Wolf-Hirschhorn	1
Mosaic 47,XYY/46,XY	1
Trisomy 13	5
Trisomy 18	1
Trisomy 21	3
STORCH, n = 13	
Congenital syphilis	2
Congenital toxoplasmosis	6
Rubella	0
Congenital cytomegalovirus	4
Congenital hepatitis B	1
Birth defects (isolated, multiple nonsyndromic), n = 38	
Anencephaly and rachischisis	3
Congenital heart defect	7
Craniosynostosis	1
Gastroschisis	2
Hydranencephaly and hydrocephaly	4
Microcephaly, constitutional or familial	11
Multiple malformations of unknown cause	8
Partial agenesis of the corpus callosum	1
Cleft palate	1
Diseases, maternal conditions, or problems at delivery, n = 27	
Maternal alcoholism or drug use	7
Hypoxic encephalopathy or acute fetal distress at birth	5
Maternal ossifying myositis	1
Maternal hyperthyroidism	1
Pregnancy-induced hypertension with or without pre-eclampsia	9
Maternal chronic arterial hypertension	2
Maternal tuberculosis	1
Maternal epilepsy	1
Other genetic diseases or other specific syndromes of the infant, n = 16	
Crouzon syndrome	1
Septo-optic dysplasia	1
Holoprosencephaly	8
Cystic fibrosis	1
Roberts syndrome (possible)	1
Meckel Gruber syndrome (possible)	1
Aicardi Goutières syndrome (probable)	1
Russel Silver syndrome	1
Familial syndrome not specified	1
Newborn with microcephaly with subsequent normal head circumference and no other findings, n = 99†	
Term newborns‡	86
Premature newborns	13
Total excluded cases	204

*STORCH, syphilis, toxoplasmosis, rubella, cytomegalovirus, and hepatitis B. Note that Costa Rica does not include hepatitis B in its standard evaluations. †Newborns that do not belong to any other category of causes.  ‡Includes 56 with and 30 without intrauterine growth restriction.

For 79% (193/243) of newborns, >1 ZIKV laboratory test was performed by urine or serum rRT-PCR or by serum IgM ELISA. Cerebrospinal fluid from 4 infants was available for testing. Serologic tests for other congenital infections were completed for syphilis (65%, 159/243), rubella (87%, 211/243), cytomegalovirus infection (69%, 168/243), and toxoplasmosis (68%, 166/243).

Among infants with suspected ZBD, 68% (165/243) underwent head ultrasonography or computed tomography (CT), 56% (136/243) underwent indirect ophthalmologic evaluation, 46% (113/243) had hearing screened by otoacoustic emissions, and 31% (75/243) underwent auditory brain response testing. Diagnostic auditory brain response testing was also performed for those with confirmed and probable cases.

### Confirmed and Probable Cases of ZIKV-Associated Birth Defects

**Figure 3:**
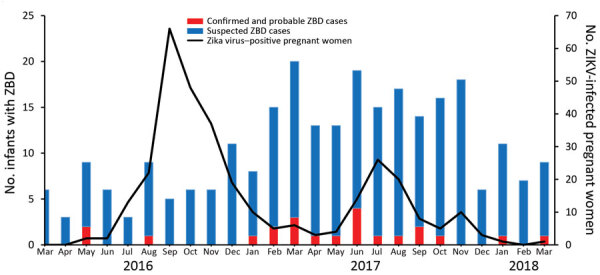
Distribution of infants with reported ZBD and pregnant women with Zika virus infection, by month, Costa Rica, March 2016–March 2018. The peak of Zika virus infection among pregnant women occurred in September 2016; the highest number of suspected cases of ZBD occurred 6 months later, March–October 2017. ZBD, Zika virus–associated birth defects.

The prevalence of ZBD during March 2016–March 2018 was 15.3 (95% CI 8.9–21.7) cases/100,000 live births, based on 22 confirmed and probable cases among 143,930 live births (Figure 3). Proportion of deaths within the first year of life among infants with ZBD was 13.6% (3/22). All death cases were classified as probable. These infants had cerebral anomalies, microcephaly, hypertonia, multiple joint contractures (n = 2), and optic nerve hypoplasia (n = 1), and were born to immigrant mothers from highly ZIKV-endemic areas.

Most infants with ZBD were full-term newborns (95%, 21/22) and had weight appropriate for gestational age (68%, 15/22). The average weight (± SD) at birth was 2,818 g (± 657 g, range 1,560–3,940 g), and height was 44.2 cm (± 3.1 cm, range 41–53 cm). Eleven infants classified as confirmed cases were positive for ZIKV (9 by IgM ELISA and 2 by IgM ELISA and rRT-PCR; Figure 2); among 11 infants classified as having probable cases, 7 had negative test results (4 by rRT-PCR and IgM ELISA and 3 by rRT-PCR alone) and 4 did not undergo laboratory testing.

**Figure 4:**
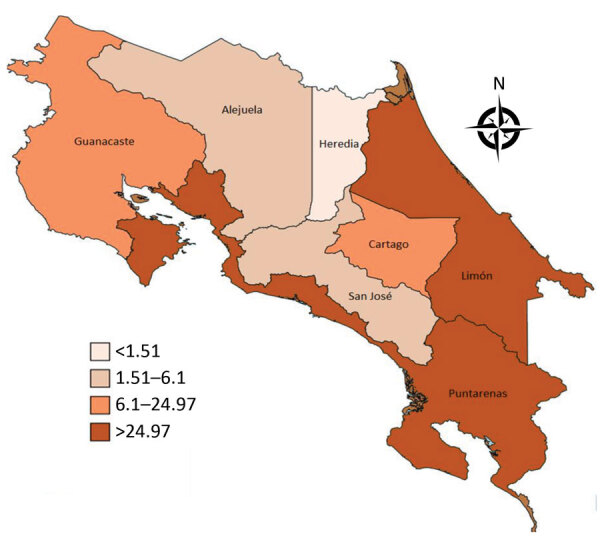
Prevalence of Zika-virus–associated birth defects (no. cases/100,000 live births), by province, Costa Rica, March 2016–March 2018. Cases are distributed by place of residence of the mother, not by place of birth. The 2 provinces in which prevalence of Zika virus–associated birth defects was highest (Puntarenas and Limón) are on the coast and have a humid tropical climate.

The provinces registering the highest prevalence of ZBD were Limón (58.8 cases/100,000 live births) and Puntarenas (37.1 cases/100,000 live births) (Figure 4). Among mothers of infants with ZBD, 64% had symptoms, 27% during the first trimester and 37% during the second trimester; 23% of the mothers had positive ZIKV rRT-PCR results during pregnancy (Table 2). Among infants with ZBD, 91% (20/22) had microcephaly (Table 3); onset was postnatal for 9% (2/22 cases). Two infants who did not have microcephaly were born to mothers who had confirmed ZIKV infection during the second trimester of pregnancy. One of these infants had cortical atrophy seen with head ultrasonography, scarring of the macula, strabismus, central hypotonia and peripheral hypertonia, swallowing difficulties, epilepsy and global neurodevelopmental delay; the other infant had normal brain images and neurodevelopment, but atrophic scarring involved the macula of both eyes.

**Table 2:** Distribution of confirmed and probable ZIKV-associated birth defects, by trimester of infection for presence of ZIKV symptoms and laboratory confirmation for the mother, Costa Rica, March 2016–March 2018*

Cases	Mothers by trimester of symptom onset									Asymptomatic mothers, subtotal	Total
	ZIKV positive through rRT-PCR of maternal sample					Without laboratory evidence in maternal sample					
	Trimester			Subtotal		Trimester			Subtotal		
	I	II	III			I	II	III			
Confirmed†	2	1	0	3		4	2	0	6	2	11
Probable‡	0	2	0	2		0	3	0	3	6	11
Total, no. (%)	2 (9)	3 (14)	0	5 (23)		4 (18)	5 (23)	0	9 (41)	8 (36)	22 (100)

*Samples were taken only from pregnant women with symptoms. rRT-PCR, real-time reverse transcription PCR; ZIKV, Zika virus. †Designated as an infant with ZIKV-associated birth defects (clinical criteria), who was positive for ZIKV by rRT- PCR or IgM-ELISA in a sample taken before hospital discharge (laboratory criteria) and had an epidemiologic link (mother with ZIKV symptoms or positive rRT-PCR during pregnancy or from a high ZIKV-endemic community). ‡Designated as an infant with ZIKV-associated birth defects who was negative for ZIKV by rRT-PCR or IgM-ELISA or was not tested but whose mother had laboratory-confirmed ZIKV infection or had symptoms compatible with ZIKV infection or had a clear exposure to ZIKV during pregnancy and no other cause that could explain the birth defect.

**Table 3:** Cases of Zika virus–associated birth defects and neurodevelopmental abnormalities, Costa Rica, March 2016–March 2018*

Clinical and neuroimaging features	No. (%) cases		
Confirmed, n = 11	Probable, n = 11	Total, n = 22
Brain defects	9 (82)	9 (82)	18 (82)
Ventriculomegaly/Hydrocephaly	8 (73)	4 (36)	12 (55)
Intracranial calcifications	8 (73)	3 (27)	11 (50)
Cerebral atrophy	4 (36)	6 (55)	10 (45)
Corpus callosum abnormalities	4 (36)	4 (36)	8 (36)
Abnormal cortical formation	3 (27)	3 (27)	6 (27)
Cerebellar abnormalities	2 (18)	0	2 (9)
Porencephaly	0	1 (9)	1 (5)
Other	0	2 (18)	2 (9)
No brain defects	2 (18)	2 (18)	4 (18)
Eye anomalies	5 (45)	4 (36)	9 (41)
Chorioretinal scarring in the macula	4 (36)	2 (18)	6 (27)
Optic nerve	3 (27)	2 (18)	5 (23)
Other	0	0	0
No eye anomalies	6 (55)	5 (45)	11 (50)
No data reported†	0	2 (18)	2 (9)
Microcephaly	11 (100)	9 (82)	20 (91)
Severe	9 (82)	5 (45)	14 (64)
Mild–moderate	2 (18)	4 (36)	6 (27)
No microcephaly	0	2 (18)	2 (9)
Hearing abnormalities, ABR evaluation	2 (18)	0	2 (9)
Sensorineural hearing loss	2 (18)	0	2 (9)
No hearing abnormalities	7 (64)	6 (55)	13 (59)
Not evaluated by ABR‡	2 (18)	5 (45)	7 (32)
Neurodevelopmental abnormalities	11 (100)	9 (82)	21 (95)
Body tone abnormalities	10 (91)	8 (73)	18 (82)
Possible developmental delay§	10 (91)	8 (73)	18 (82)
Possible visual impairment	8 (73)	4 (36)	12 (55)
Congenital contractures	5 (45)	5 (45)	10 (45)
Seizures, excluding febrile	7 (64)	1 (9)	8 (36)
Movement abnormalities	6 (55)	5 (45)	11 (50)
Swallowing abnormalities	6 (55)	3 (27)	9 (41)
No abnormalities	0	1 (9)	1 (5)
No data reported†	0	1 (9)	1 (5)

*ABR, auditory brain response test. †These infants had a normal result for newborn hearing screening by otoacoustic emissions testing and were not evaluated by ABR because they were lost to follow-up. Three infants did not have any hearing screening because they died soon after birth. ‡Includes infants for whom an evaluation was not performed or records were not obtainable. §For 4 children (1 with a confirmed case and 3 with a possible case), developmental delay was not evaluated by any specific method.

Head ultrasonography was performed for 21 of the 22 infants classified as confirmed and probable ZBD (6 of them also underwent CT, magnetic resonance imaging, or both), and the other underwent head CT. Among these infants, 82% (18/22) had evidence of >1 brain defect. Those without brain defects evident by imaging had defects in the eye, body tone, or neurodevelopment.

Ophthalmologic evaluation was performed for 91% (20/22) of infants classified as having confirmed and probable cases; eye anomaly was detected for 45% (9/20). Sensorineural deafness was present in 9% (2/22); however, only 15/22 (68%) underwent audiologic evaluation with diagnostic auditory brain response testing. At least 1 neurodevelopmental anomaly was present in 95% (21/22) of infants; most (82%) had body tone anomalies (mainly hypertonia or spasticity) and possible neurodevelopmental delay. Other frequent manifestations included multiple contractures, seizures, movement anomalies, swallowing anomalies, and possible visual impairment (strabismus, nystagmus, or failure to fix and follow).

## Discussion

In Costa Rica, most infants with ZBD were born ≈1 year after the onset of autochthonous circulation of ZIKV and 6 months after peak incidence of ZIKV infection among pregnant women. Similar findings have been observed in Brazil, Colombia, and the United States, where the peak incidence was observed ≈6 months after the ZIKV epidemic, corroborating a temporal link between ZIKV infection and associated birth defects (*7*,*23*,*24*). Most infants with ZBD were born to mothers who reported symptoms in the first and second trimesters of pregnancy (64%), consistent with other reports (*25*–*29*); however, because 36% were born to mothers who were asymptomatic, the proportion of infections in early to mid-pregnancy is probably greater.

The birth prevalence of infants with confirmed and probable ZBD during the enhanced surveillance period was 15.3 (95% CI 8.9–21.7) cases/100,000 live births. Birth prevalence of microcephaly, which was monitored before the ZIKV outbreak in Costa Rica, increased by almost 4-fold after the ZIKV outbreak, from 4.2 cases/10,000 live births to 15.5 cases/10,000 live births. Although we recognize that the birth prevalence of this defect may be underreported during non–ZIKV-epidemic times, these data are consistent with the experience in other countries, where the prevalence of microcephaly increased >4-fold (Colombia, French Polynesia, United States) (*7*,*24*,*25*) and up to 9-fold (Brazil) (*30*,*31*). Heightened awareness of the possible association between congenital ZIKV infection and microcephaly, as well as country-specific protocols to improve identification of this traditionally underascertained birth defect, probably contributed to increased prevalence estimates (*32*). Additional efforts were necessary to differentiate microcephaly as a component of ZBD from microcephaly from other causes in Costa Rica and in other ZIKV-affected locations. Consistent with published case series from Brazil (*11*,*12*) and from the US Zika Pregnancy and Infant Registry (*26*), we found that the most frequent clinical finding was microcephaly and most cases were classified as severe. The percentage of brain abnormalities was similar to what has been published (*11*,*26*,*33*), and among infants who underwent neuroimaging, the most common findings were ventriculomegaly and cerebral calcifications, consistent with results of a recent meta-analysis (*34*). Two case-patients did not have microcephaly; both were born to mothers who had confirmed ZIKV infection during the second trimester of pregnancy, and both had other clinical findings consistent with congenital ZIKV infection. These findings have also been reported in studies from Brazil (*33*), where up to 1 in 5 infants with confirmed or probable ZBD had a normal head circumference (*12*).

Anomalies of the eye have also been associated with congenital ZIKV infection. Among the infants with eye anomalies in our study, most common were anomalies of the fundus, primarily chorioretinal scars or abnormal macular pigmentation and papillary/optic nerve atrophy. Several case series reported the same findings for 24%–55% of case-patients (*9*), mainly children of mothers infected with ZIKV during the first trimester (*35*,*36*). ZIKV-associated eye defects were found without microcephaly in 10/24 (42%) infants born to mothers with rRT-PCR–confirmed ZIKV infection during pregnancy; 8 (33%) of these infants had no abnormal brain findings (*36*), consistent with what we found for 1 infant with a confirmed case.

Sensorineural deafness was detected in 13% of infants by diagnostic auditory brain response testing. One study that specifically evaluated hearing loss in children with birth defects found sensorineural deafness in 6% by using auditory brain response testing (*37*); most cases also had neurodevelopmental anomalies previously described in the literature (*9*–*12*,*26*,*38*). The most frequent neurodevelopmental anomalies were tone abnormalities (primarily hypertonia), movement anomalies, and congenital joint contractures. Some neurologic and developmental alterations associated with microcephaly are secondary to CNS damage caused by ZIKV. Described in our case series and in other studies, these alterations include movement abnormalities and posturing (50%), swallowing abnormalities (41%), and epilepsy (36%) (*39*).

Our descriptive analysis is subject to limitations. Findings are based on a passive surveillance system enhanced with confirmation of the diagnosis; thus, information depends on the completeness of reporting, case ascertainment, and workup of suspected cases to verify microcephaly and determine which cases are probably ZIKV associated. The NBDSS collects data on live births only; findings are not generalizable to stillbirths and miscarriages. Comparing the prevalence of ZBD among countries is difficult because surveillance system methods and definitions of microcephaly and suspected cases vary and evaluations and criteria used to define ZBD might differ substantially (*40*). Another consideration is the known limitations of ZIKV laboratory tests (*41*–*43*). Among infants with ZBD, 50% had a positive ZIKV laboratory test result, all by ZIKV IgM ELISA and only 1 by rRT-PCR. Nonspecific reactivity resulting in a false-positive IgM result might have led to misclassification of cases as confirmed; however, false-positive IgM results seem unlikely, given the timing of ZIKV testing in these infants. Eleven infants with ZBD but without laboratory evidence were classified as probable cases. Of these, 7 had negative results by rRT-PCR, IgM ELISA, or both. The low detection of laboratory evidence for ZIKV infection in these infants probably reflects recognized challenges of laboratory testing, including the unknown sensitivity and specificity of testing of infants (*41*,*42*). In addition, given possible cross-reactivity for other flaviviruses and the need to prioritize resources, we did not conduct plaque-reduction neutralization tests. To help address laboratory limitations, we used the combination of clinical, epidemiologic, and laboratory data to classify cases. However, we cannot exclude an alternate etiology for birth defects in infants classified as probable cases.

The Pan American Health Organization recommends surveillance of ZIKV disease in pregnant women and monitoring outcomes of infants born with brain anomalies (*44*). Many countries have implemented surveillance to monitor infants of ZIKV-positive mothers to capture cases of ZBD, to determine the risk for birth defects, and to examine neurodevelopmental anomalies (*25*,*30*,*32*). Population-based birth defects surveillance programs along with monitoring pregnant women with ZIKV disease provide an example of a complementary approach to ascertaining exposures and outcomes to better monitor new and emerging threats during pregnancy and effects on infants (*45*). Costa Rica National Guidelines established laboratory sampling and monitoring of every child born to symptomatic women (*46*). In our analysis, 23% of mothers of infants with confirmed and probable ZBD had a positive laboratory test result for ZIKV (Table 2); had the enhanced birth defects surveillance system not been implemented, 77% of cases would not have been linked to ZIKV. In addition, 60%–80% of ZIKV infections are asymptomatic, and in Costa Rica, the laboratory test for ZIKV is performed only for symptomatic pregnant women. Given these challenges, the benefit of combining an intensified birth defects surveillance system with surveillance of pregnant women with laboratory-confirmed ZIKV infection, as was done in Costa Rica and Colombia (*29*), is very useful, especially for countries with few resources.

The success of surveillance for ZBD in Costa Rica depended on the strict application of standard operating procedures and the active participation of healthcare personnel to enhance ascertainment of component anomalies, such as microcephaly, and to identify infants with sufficient evidence of a confirmed or probable ZIKV etiology for their birth defects. Thus, global establishment and strengthening of NBDSS is essential, as recommended by the World Health Organization at its 63rd World Health Assembly (Resolution WHA63.17, https://apps.who.int/gb/ebwha/pdf_files/WHA63-REC1/WHA63_REC1-en.pdf). Microcephaly is not the only congenital anomaly that should be monitored after ZIKV infection; other birth defects, such as congenital brain and eye defects and joint contractures, should also be monitored. Monitoring children born to ZIKV-positive mothers and those with ZBD or neurodevelopmental anomalies through at least the first years of life can increase identification of additional associated abnormalities such as deafness, eye or vision anomalies, postnatal onset of microcephaly, and substantial neurodevelopmental abnormalities. Other neurodevelopmental disabilities might become apparent after 1 year of age; thus, following children to 3 years of age is valuable and may enhance surveillance of ZBD and neurodevelopmental outcomes in Costa Rica.
